# Exploration of *Lactiplantibacillus fabifermentans* and *Furfurilactobacillus rossiae* as potential cocoa fermentation starters

**DOI:** 10.1111/jam.15687

**Published:** 2022-07-02

**Authors:** Dea Korcari, Giovanni Ricci, Alberto Fanton, Davide Emide, Alberto Barbiroli, Maria Grazia Fortina

**Affiliations:** ^1^ Dipartimento di Scienze per gli Alimenti, la Nutrizione e l'Ambiente Università degli Studi di Milano Milan Italy; ^2^ Rizek Cocoa S.A.S. San Francisco de Macorìs Dominican Republic

**Keywords:** antifungal activity, Dominican cocoa bean, fermentation, LAB, proteolysis, starter cultures, stress resistance

## Abstract

**Aims:**

To investigate the characteristics of two minority autochthonous LAB species, with particular regard to those properties that could be exploited in an improved cocoa fermentation process from a quality and safety point of view.

**Methods and Results:**

Bacterial, yeast and mould strains characteristic of spontaneously fermented Dominican cocoa beans were isolated and identified by 16S or 26S rRNA gene sequencing. The potential of two autochthonous strains of LAB belonging to the species *Lactiplantibacillus fabifermentans* and *Furfurilactibacillus rossiae* were investigated. The two selected LAB strains were able to utilize glucose and fructose, produced mainly D‐L lactic acid and had a good ability to resist to cocoa‐related stress conditions such as low pH, high temperature and high osmotic pressure, as well as to grow in sterile cocoa pulp. The strains did not inhibit the growth of yeasts and acetic acid bacteria, that are essential to the cocoa fermentation process, and possessed a complex pool of peptidases especially active on hydrophobic amino acids. The strains also showed antifungal activity against mould species that can be found at the final stages of cocoa fermentation, as *Aspergillus tamarii, A. nidulans, Lichtheimia ornata* and *Rhizomucor pusillus*.

**Conclusions:**

The tested strains are good candidates for the design of starter cultures for a controlled cocoa fermentation process.

**Significance and Impact of the Study:**

This research showcases the potential of two alternative LAB species to the dominating *Lactiplantibacillus plantarum* and *Limosilactibacillus fermentum* as cocoa fermentation starters, with an interesting activity in improving the safety and quality of the process.

## INTRODUCTION

Cocoa bean fermentation is a crucial step in the production process of chocolate and its quality. This step is fundamental in determining the flavour profile and composition of chocolate, and in reducing the excessive bitterness and astringency of unfermented cocoa. At industrial level cocoa fermentation is typically a spontaneous process carried out with different methodologies such as in heaps, boxes or trays, with banana or plantain leaves sometimes used to contain the cocoa.

The variable nature of fermented cocoa is to this day a major problem for the cocoa and chocolate producing industries. With all other factors remaining unchanged, the outcome of the spontaneous fermentation is still difficult to control, and this is a drawback both for chocolate makers that need to use cocoa blends to maintain the uniformity of their recipes, and for cocoa producing companies for which the variable quality is often translated in an economic loss. Although the understanding of the cocoa fermentation process and the setup of controlled fermentations have been an important step toward a more controlled process, a wider understanding of the impact of the different bacterial species on the fermentation is needed to design starter cultures that have a positive role in the flavour and quality of cocoa (Calvo et al., 2021).

Cocoa beans inside the cocoa pod are sterile, and the microbial species that conduct the fermentation derive from environmental contaminations such as the surface of the pods, from the hands and machetes used to open them, from the sacks and baskets used for transportation, from fermentation boxes and plantain leaves (De Vuyst & Weckx, 2016).

The fermentation is carried out by yeasts and lactic acid bacteria (LAB) in the initial anaerobic stage. Glucose, fructose and citric acid are fermented into organic acids and ethanol by both LAB and yeasts. Yeasts are also able to convert the pulp pectin in simple sugars thanks to their pectinolytic activity. After 1–2 days the fermenting mass is mixed, a process that introduces oxygen and causes a shift in the microbial population: whereas the presence of yeasts and LAB decreases, aerobic acetic acid bacteria (AAB) begin to develop and convert ethanol and residual sugars into acetic acid. This phase is characterized by an increase in temperature that in synergy with the presence of acetic acid kill the embryo, activating its hydrolytic enzymes and inhibiting the germination. At the end of the fermentation spore forming *Bacillus* species and mould may grow.

Independently of the region of cultivation, the dominating LAB species in cocoa fermentation are facultatively heterofermentative *Lactiplantibacillus plantarum* and strictly heterofermentative *Limosilactibacillus fermentum* (Schwendimann et al., 2015). Other species such as *Fructobacillus* spp. and *Leuconostoc* spp. have been reported to play an important role at the beginning of the fermentation, however, previous starter culture design experiments for cocoa fermentation have focused on the former two species (De Vuyst & Weckx, 2016).

The goal of this work was to evaluate the potential of two autochthonous strains of LAB belonging to the species *Lactiplantibacillus fabifermentans* and *Furfurilactibacillus rossiae*, isolated from fermented cocoa, to be used as adjunct cultures for an improved cocoa bean fermentation process.

These species are not considered to be dominant in cocoa fermentations. Indeed, the facultatively heterofermentative *L. fabifermentans* first described by De Bruyne et al. (2009) from Ghanaian cocoa fermentations, was reported as a minority species at the first stages of Ecuadorian cocoa fermentation (Papalexandratou et al., 2011), and represented only 1.23% of the LAB isolated from fermented cocoa in the Côte d'Ivoire (Adiko et al., 2018), although, because of the high similarity with *L. plantarum*, this species may have been underestimated in previous cocoa fermentation microbiota research. *L. fabifermentans* has one of the biggest genomes of LAB which shows a great genomic versatility, with a wide range of carbohydrate utilization (Campanaro et al., 2014). The reported preference for fructose, rather than glucose, can reduce the competition with yeast, making this species a good candidate for a mixed starter culture (Lefeber et al., 2011).

*F. rossiae*, on the other hand, has never been described in cocoa fermentations before, to the authors' knowledge. This obligately‐heterofermentative LAB has been isolated from a wide range of environmental niches, such as sourdoughs, fruit, fermented meat, and animal and human gut. This species can metabolize a substantial number of carbohydrates, and genomic research has shown its potential for polysaccharide degradation, as revealed by an in‐silico analysis (De Angelis et al., 2014).

This research aims to investigate some characteristics of these minority, scarcely studied species, with particular regard to those properties that could be exploited in an improved cocoa fermentation process from a quality and safety point of view.

## MATERIALS AND METHODS

### Strain isolation and maintenance

Microbial population was isolated from a sample of cocoa of the Criollo variety provided by Rizek Cacao S.A.S., at different fermentation times. The LAB isolation was performed by a culture‐dependent method, plating adequate dilutions in Man Rogosa Sharpe (MRS‐Difco Lab) agar plates, incubated at 30°C for 48 h. For each fermentation day, approximately 10 colonies were isolated. Pure cultures of each strain were routinely subcultured in MRS broth at 30°C for 24 h.

Similarly, yeast strains were isolated by plating adequate dilutions in Yeast extract Glucose Chloramphenicol (YGC) agar plates (MilliporeSigma) incubated at 28°C for 72 h. Isolated strains were maintained in Yeast extract Peptone Dextrose (YPD) broth incubated at 30°C for 24 h. The composition of the YPD medium is (g L^−1^): yeast extract 10, glucose 20, peptone 20.

Four mould isolates were obtained from a contaminated batch of cocoa at the end of the fermentation period. For isolation YGC plates were used. Plates were incubated at 28°C for 5–7 days. Moulds were isolated and transferred to Malt Extract Agar (MEA) plates (Thermo Fisher Scientific) for their maintenance and identification. In this study we also used other mould strains previously isolated from fermented food and identified in our laboratory, as *Aspergillus flavus, Aspergillus niger, Mucor circinelloides* and *Fusarium verticillioides*. Spores were collected by pouring sterile distilled water containing 9 g L^−1^ NaCl on the plates after complete sporification, slowly agitating and storing in sterile tubes.

All isolates were deposited in the culture Collection of the Department of Food, Environmental and Nutritional Sciences, University of Milan, Italy, at −80°C in their maintenance medium, with the addition of 150 ml L^−1^ glycerol.

*L. fabifermentans* SAF13, *F. rossiae* SAF51, *L. plantarum* B7 and *Saccharomyces cerevisiae* TB2.3 were selected for further studies.

*Acetobacter pasteurianus* DSM 3509 was used for co‐culture experiments. The strain was maintained in Glucose Yeast extract Calcium Carbonate (GYC) agar plates incubated at 28°C for 48 h. The composition of the GYC agar medium is as follows (g L^−1^): glucose 50, yeast extract 10, calcium carbonate 30, agar 15 (MilliporeSigma).

### Molecular identification of the isolates and q‐PCR experiments

The identification of yeast isolates was performed by 26S rRNA gene sequencing or ITS amplification and restriction with restriction enzymes *Hind*III and *Hinf*I, as previously reported (Decimo et al., 2017). Mould identification was carried out by 26S rRNA gene sequencing (White et al., 1990).

LAB isolates were identified by 16S rRNA gene sequencing. For the subsequent detection of *F. rossiae* and *L. fabifermentans* both in single culture and in co‐cultures, species‐specific probes were used or designed. For the detection of *F. rossiae*, primers designed by Riedl et al. (2017) were used. For the detection of *L. fabifermentans*, primers were designed in this study using the 16S rRNA gene deposited in the GeneBank database and aligning to the 16S rRNA genes of the affine *L. plantarum* species to check for specificity. Furthermore, the melting temperature (T_m_) was optimized to avoid mismatches. The primers and thermal cycles are reported in Table 1. For qPCR experiments, calibration curves were designed by inoculating decimal dilutions of an overnight grown culture of each *F. rossiae* and *L. fabifermentans* strain in a range from 3–8 log cycles in MRS broth. Cell lysis of the bacterial pellet recovered from MRS and DNA extraction was performed as reported by Mora et al. (2000).

**TABLE 1:** Primers and thermal cycles for species‐specific PCR analyses

Species	Primer sequences	Thermal cycle	Reference
*Lactiplantibacillus fabifermentans*	F: CTGGTATTGATTGGTACTTGT R: ACCTCACCATCTAGCTAATG	95 °C × 10 s 59 °C × 20 s 72 °C × 20 s	This study
*Furfurilactibacillus rossiae*	F: GGCGTGCCTAATACATGCAA R: TGTCTCGTCAATCTGGTGCAA	95 °C × 10 s 60 °C × 20 s 72 °C × 20 s	Riedl et al., 2017

The PCR reaction was carried out in a total volume of 15 μl, containing 7.5 μl of qPCR mix (SSO Fast Supermix, BioRad, Hercules, USA), 0.36 μl of each primer (0.3 μmol L^−1^), 1.78 μl of PCR grade water and 5 μl of DNA. The efficiency of the standard curves was in the range 90%–110% and the R^2^ was >0.99.

### Carbon source utilization and organic acids production

The growth of the strains in presence of glucose, fructose and sucrose was tested in MRS broth containing 10 g L^−1^ of each sugar. Overnight grown cultures of each strain were twice washed with sterile saline water and inoculated in 200 μl of medium at a concentration of 10^6^ CFU ml^−1^ in 96 well plates. The growth was measured using an automated microplate reader (Eon™ Microplate Spectrophotometer, BioTek) at 600 nm. The lag time was calculated by the instrument.

The production of acetic acid and lactic acid after 24 h incubation at 30°C was measured using commercial assay kits according to the manufacturer's instructions (R‐Biopharm AG).

### Tolerance to stress conditions

The ability of *L. fabifermentans* SAF13 and *F. rossiae* SAF51 to resist to different stress conditions were tested in MRS broth at different temperatures (from 25 to 50°C) and pH values (from 3.0 to 6.5). The tolerance to high osmolarity was evaluated in MRS broth containing 150 g L^−1^ and 300 g L^−1^ glucose or fructose.

The growth was evaluated by measuring the Optical Density at 600 nm (OD_600nm_), after 48 h of incubation. The autochthonous *L. plantarum* strain B7 was used as comparison.

### Autolytic activity in buffer system

The rate of autolysis was determined according to the method described by Ayad et al. (2004) and Ma et al. (2011). The cell pellets were resuspended in citrate buffer (0.1 mol L^−1^pH 4.0) containing 0.5 mol L^−1^ NaCl and then diluted to OD 650 nm = 1.0. The rate of autolysis was determined as absorbance decrease at 650 nm after incubation at 30°C for 48 h, using the formula %autolysis = (1−OD_48h_/OD_t0_) *100.

### Proteolytic activity

The proteolytic (aminopeptidase, dipeptidyl peptidase and endopeptidase) activity of *L. fabifermentans* SAF13 and *F. rossiae* SAF51 was characterized in comparison to *L. plantarum* B7.

A cellular pellet of the three strains was recovered from an overnight grown culture in MRS medium by centrifugation at 8000 × *g* for 10 min, 4°C. The pellet was washed twice, resuspended in physiological solution (9 g L^−1^ NaCl), and then the cells lysed in a TissueLyzer (Qiagen, Hilden, Germany) at 30 s^−1^ for 3 min. Cellular debris were removed by centrifugation at 13000 × *g* for 15 min, 4°C, and the clear supernatant (cell lysate) was stored at −20°C until use. The protein content of cell lysates was quantified by a dye‐binding method (Bradford, 1976), whereas the protein pattern was characterized by SDS‐PAGE (Laemmli, 1970).

Proteolytic activities were determined by using the following synthetic p‐nitroanilide (pNA) substrates: Leu‐pNA, Met‐pNA, Ala‐pNA, Pro‐pNA, Lys‐pNA for aminopeptidase activities; Gly‐Pro‐pNA for prolylpeptidases activity; N‐Benzoyl‐Arg‐pNA (BAPA) and N‐Suc‐Ala‐Ala‐Pro‐Phe‐pNA (SUNA) for trypsin‐ and chymotrypsin‐like, respectively, endoprotease activities (Merck Life Science S.r.l., Milano, Italy). Activity assays were adapted from Iametti et al. (2002). The pNA substrate solutions were prepared at the concentration of 0.5 mmol L^−1^ in sodium acetate buffer (50 mol L^−1^, pH 4.5). The reactions were started by adding 0.2 ml cell lysate (diluted at about 0.4 mg ml^−1^ protein concentration) to 0.8 ml substrate solution. The pNA released in 60 min at 37°C was determined spectrophotometrically at 405 nm. One unit of specific activity was defined as a 0.001 increment in absorbance per hour per amount of protein (mg), under the assay condition.

### Antifungal activity assay

Antifungal activity against *Aspergillus flavus, Aspergillus niger, Aspergillus tamarii, Aspergillus nidulans, Lichtheimia ornata, Rhizomucor pusillus. Mucor circinelloides* and *Fusarium verticillioides* was tested using the overlay method (Axel et al., 2015). Each LAB strain tested was streak plated in MRS agar and grown at 30°C for 72 h. Afterwards a layer of MEA soft agar (7 g ml^−1^ agar) containing 10^4^ ml^−1^ spores suspension was slowly added to the plates that were incubated at 30°C for 3 days. Antifungal activity was evaluated as clear zones of inhibition around the bacterial smears.

### Evaluation of growth in cocoa

The bacterial growth ability was evaluated in cocoa pulp. For this purpose, cocoa pods were harvested, and the surface was cleaned with alcohol. The pulp was collected in sterile conditions and was pasteurized (65°C for 30 min), to ensure the absence of contamination. The sterility was confirmed by plating in MRS agar, YPD agar and PCA and incubating at 30°C for 48 h. Approximately 10^5^ CFU ml^−1^ of each LAB strain tested were inoculated in the cocoa pulp and the growth was evaluated by dilution and plating in MRS agar after 16 and 40 h of incubation at 30°C.

### Growth in co‐culture

To investigate the possibility of *F. rossiae* SAF51 and *L. fabifermentans* SAF13 strains to be used in conjunction with one another, co‐culture assays were set up. The growth was performed in MRS broth by inoculating 10^5^–10^6^ CFU ml^−1^ of each strain and incubating at 30°C for 16 and 40 h. The growth of each strain was evaluated by qPCR.

The two LAB strains were also grown in conjunction with the autochthonous strain *S. cerevisiae* TB2.3. The growth of the yeast was evaluated with the plate count method in YGC agar and the growth of bacterial strains was evaluated with a qPCR assay as previously described.

Co‐cultures of LAB strains and *A. pasteurianus* DSM 3509 were carried out by inoculating 10^6^ CFU ml^−1^ of cells in MRS broth in agitation at 150 rpm and 28°C. The growth of LAB was evaluated by plate count in MRS agar incubated in anaerobic conditions, the growth of *A. pasteurianus* DSM 3509 was evaluated in basal MRS agar medium without glucose and with 40 ml L^−1^ ethanol as sole carbon source.

### Statistical analysis

All results are expressed as mean ± SD of three independent replicates of each experiment. Analysis of variance (one‐way ANOVA; *p* < 0.05) was performed by using SigmaPlot version 14.0 (Systat Software Inc.,). Differences were considered statistically significant for *p* < 0.05.

## RESULTS

### Microbial population composition

The LAB and yeast populations isolated from the Dominican cocoa fermentation are reported in Table 2. The LAB population had a larger diversity in the first 48 h of fermentation. After 72 h, in correspondence with the mixing of the cocoa mass, the biodiversity of LAB was significantly reduced, and the population was comprised of mainly *Lactiplantibacillus plantarum*, that was present throughout all days of fermentation. One *L. plantarum* strain, strain B7 was chosen as representative of the dominant species already adapted to cocoa fermentation habitat and used in these comparative studies. Apart from other species commonly associated with fermented cocoa, such as *Lacticaseibacillus paracasei* and *Levilactobacillus brevis*, minority species such as *Lacticaseibacillus rhamnosus*, *Furfurilactibacillus rossiae*, *Lactiplantibacillus fabifermentans* and *Liquorilactobacillus satsumensis* were also isolated. We focused the attention toward the minority *F. rossiae*, and *L. fabifermentans* species, since their presence in fermented cocoa and/or in fermented food has been demonstrated but poorly studied. The two strains *L. fabifermentans* SAF13 and *F. rossiae* SAF51 were selected for their evaluation.

**TABLE 2:** LAB and yeast species isolated at different fermentation times

Microbial species	Fermentation time (h)					Total isolates	Percentage
	24	48	72	96	120		
	Number of isolates						
LAB species							
*Lactiplantibacillus plantarum*	0	9	2	23	10	44	73.3
*Lacticaseibacillus paracasei*	0	2	0	2	0	4	6.7
*Levilactobacillus brevis*	0	1	2	0	0	3	5.0
*Lacticaseibacillus rhamnosus*	0	1	0	0	2	3	5.0
*Lactiplantibacillus fabifermentans*	0	0	3	0	0	3	5.0
*Furfurilactibacillus rossiae*	0	2	0	0	0	2	3.3
*Liquorilactobacillus satsumensis*	0	0	0	1	0	1	1.7
Yeast species							
*Saccharomyces cerevisiae*	7	14	16	9	7	53	65.4
*Torulaspora delbrueckii*	3	0	0	4	0	7	8.6
*Schizosaccharomyces pombe*	0	0	0	0	6	6	7.4
*Hanseniaspora opuntiae*	3	2	0	0	0	5	6.2
*Wickherhamomyces pijperi*	3	0	0	0	0	3	3.7
*Starmerella bacillaris*	1	2	0	0	0	3	3.7
*Pichia kudriavzevii*	2	0	0	0	0	2	2.5
*Pichia manshurica*	0	2	0	0	0	2	2.5

The dominating yeast species was *S. cerevisiae*, which is also typical of cocoa fermentations. This species represented 65% of all isolates identified. Other identified yeasts, such as *Hanseniaspora opuntiae*, *Torulaspora delbrueckii* and *Pichia* spp., are also typical of fermented cocoa. For co‐culture experiments with LAB strains, one strain of the more representative yeast of the cocoa fermentation, *Saccharomyces cerevisiae* TB2.3, was chosen.

The four mould strains isolated from a batch of mouldy cocoa belonged to the species *Aspergillus tamarii, Aspergillus nidulans, Lichtheimia ornata* and *Rhizomucor pusillus*.

### Carbon source utilization, lactic and acetic acid production and tolerance to stress conditions

*L. fabifermentans* SAF13 and *L. plantarum* B7 utilized glucose, fructose and sucrose as carbon sources, whereas *F. rossiae* SAF51 could only use glucose and fructose. Moreover, *F. rossiae* SAF51 had a significantly higher lag time (14.32 ± 0.01 h) compared to *L. plantarum* B7 (4.64 ± 0.01 h) and to *L. fabifermentans* SAF13 (3.75 ± 0.01 h), when grown either in glucose or in fructose.

After 24 h of growth, *L. fabifermentans* SAF13 and *L. plantarum* B7 produced mainly D‐L lactic acid: its final concentration (about 11 g L^−1^ L‐lactic acid and 7.5 g L^−1^ of D‐lactic acid for *L. fabifermentans*, 11 g L^−1^ L‐lactic acid and 6.3 g L^−1^ of D‐lactic acid for *L. plantarum*) was 53 times higher than the concentration of acetic acid for both strains (0.33–0.34 g L^−1^). *F. rossiae* SAF51 also produced lactic acid as the main product of its fermentation, but the proportion of acetic acid was closer to 1:2 (3.7 g L^−1^ D‐lactic acid, 4.9 g l^−1^ L‐lactic acid and 3.5 g L^−1^ acetic acid).

**FIGURE 1:**
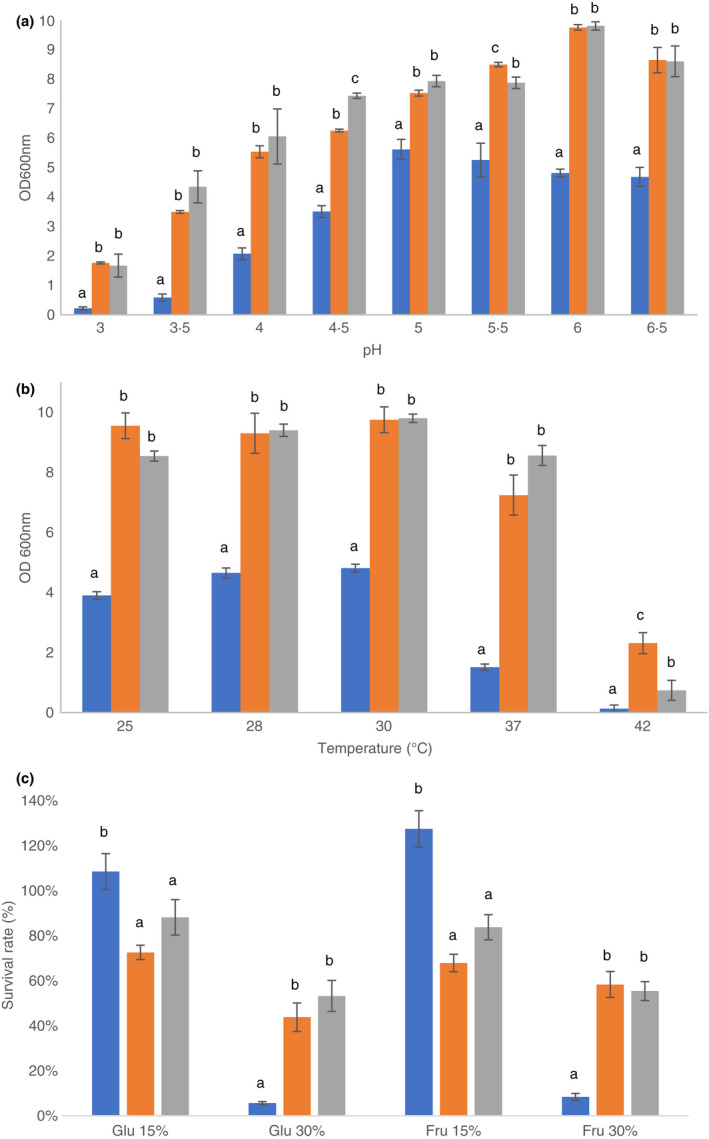
Growth at different pH (a) and temperatures (b) and survival rate relative to standard MRS medium (c) of the strains *F. rossiae* SAF51 (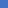 ), *L. fabifermentans* SAF13 (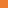 ) and *L. plantarum* B7 (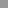 ). Different letters indicate significant differences (*p* < 0.05).

All tested strains were able to grow in cocoa‐related stress conditions such as low pH, high temperature and high osmotic pressure (Figure 1). *L. fabifermentans* SAF13 and *L. plantarum* B7 had an optimal growth at pH 6, but a moderate level of growth was also observed at pH 3; *F. rossiae* SAF51 was more sensitive to low pH values, showing very limited growth at pH 3. *L. fabifermentans* SAF13 was the most resistant strain to high temperatures, growing at temperatures up to 42°C. No growth was registered at 50°C.

### Autolysis

The autolytic phenotype of the LAB species under investigation was studied at pH 4 and 0.5 mol L^−1^ NaCl to simulate some of the conditions of cocoa pulp. The results indicate that *F. rossiae* SAF51 and *L. plantarum* B7 have a percentage of autolysis of 28.0% and 23.9%, respectively. *L. fabifermentans* SAF13 on the other hand had a very limited autolytic ability, as the OD was reduced by only 5.4%.

### Antifungal activity

The ability of the two selected strains to inhibit the fungal growth was evaluated in comparison with the strain *L. plantarum* B7.

**FIGURE 2:**
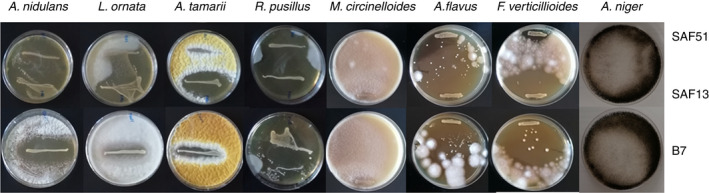
Antifungal activity overlay assay of strains *F. rossiae* SAF51, *L. fabifermentans* SAF13 and *L. plantarum* B7. Antifungal activity is represented by the clear zone surrounding the bacterial smear.

As shown in Figure 2, *L. fabifermentans* SAF13 and *L. plantarum* B7 showed inhibitory activity toward the mould species that can be found at the final stages of cocoa fermentation, particularly, *L. fabifermentans* SAF13 against *L. ornata* and *A. tamarii*. *F. rossiae* SAF51 showed no inhibition toward *L. ornata* and *A. tamarii*, whereas it could inhibit the growth of *A. nidulans*. Moreover, the three LAB tested showed interesting antifungal activity toward *A. flavus* and *F. verticillioides*, while no inhibition was evaluable for *A. niger* and *M. circinelloides*.

### Proteolytic activity

**FIGURE 3:**
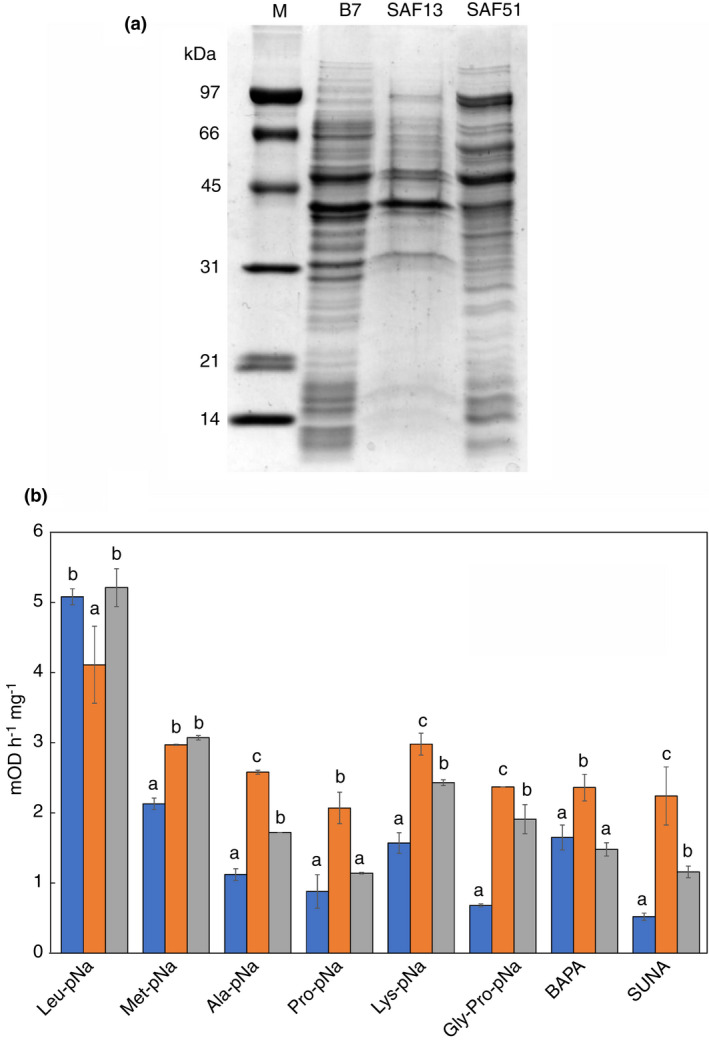
Proteasic activity of cell lysates. A: SDS‐PAGE of the cell lysate of *L. plantarum* B7, *L. fabifermentans* SAF13 and *F. rossiae* SAF51; for each sample 5 μg of protein were loaded. B: Proteolytic activity of strains *F. rossiae* SAF51 (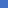 ), *L. fabifermentans* SAF13 (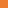 ) and *L. plantarum* B7 (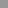 ) expressed in mOD h^−1^ mg^−1^. Different letters indicate significant differences (*p* < 0.05).

In order to evaluate the potential proteolytic activity released upon lysis, cell lysates of *L. fabifermentans* SAF13, *F. rossiae* SAF51 and *L. plantarum* B7 was produced in a bead mill. *L. fabifermentans* SAF13 cell lysate generally had a protein content three time lower than the other two strains. This evidence suggests a greater resistance of *L. fabifermentans* SAF13 also toward mechanical stress compared to *F. rossiae* SAF51 and *L. plantarum* B7, in apparent agreement with autolysis data. The three cell lysates showed peculiar protein patterns (Figure 3a), with the greatest differences observed in *L. fabifermentans* SAF13.

Proteolytic activities of the strains *L. fabifermentans* SAF13, *F. rossiae* SAF51 and *L. plantarum* B7 are shown in Figure 3b. All strains possessed a complex pool of peptidases. The highest aminopeptidase activity was observed toward the hydrophobic amino acids leucine, but significant activity was also found on alanine, methionine and lysine. In addition, iminopeptidase activity on Pro‐pNA, prolylpeptidase activity on Gly‐Pro‐pNA and endoproteolytic activity on BAPA and SUNA were also present in all the three strains. *L. fabifermentans* SAF13 had a significantly higher activity on all tested substrates except for Leu‐pNA and Met‐pNA, whereas *F. rossiae* SAF51 in general was the strain that possessed the lowest activity.

### Growth in cocoa and in co‐culture

**FIGURE 4:**
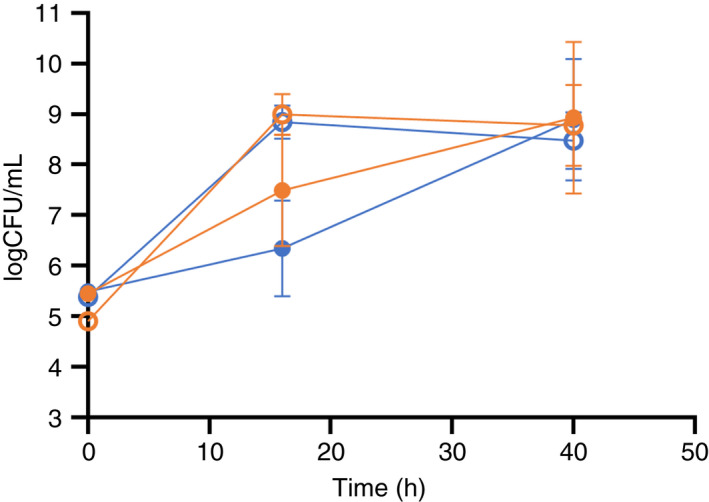
Growth in MRS (empty shapes) and cocoa pulp (full shapes) of the LAB strains *F. rossiae* SAF51 (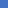 ), *L. fabifermentans* SAF13 **(** 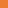 **).**

The growth of *L. fabifermentans* SAF13 and *F. rossiae* SAF51 strains in cocoa pulp is represented in Figure 4. Compared to the growth in liquid MRS broth, the growth in cocoa pulp is slower, but after 40 h the final cell concentration in the two conditions is similar.

**FIGURE 5:**
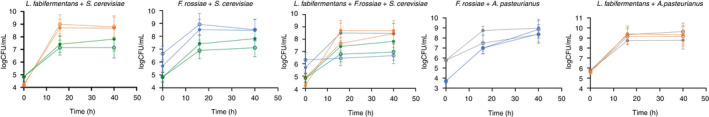
Viability of strains grown in monoculture (full shapes), or co‐culture (empty shapes) grown in MRS broth and analysed at 16 and 40 h. the two LAB strains in the three‐strain mixed culture were discriminated by qPCR. *F. rossiae* SAF51 (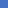 ), *L. fabifermentans* SAF13 (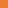 ) *S. cerevisiae* TB 2.3 (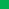 ), *A. pasteurianus* DSM 3509 (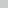 ).

Mixed culture experiments were set up in MRS broth (Figure 5). Firstly, the ability of each LAB strain to grow in association with the cocoa related yeast strain *S. cerevisiae* TB2.3 was investigated. After 16 or 40 h of incubation, the yeast growth was slightly limited by the presence of the LAB strains.

In the three‐strain combination, whereas the growth of *L. fabifermentans* SAF13 and *S. cerevisiae* TB2.3 closely resembled the growth in two‐strain co‐cultures, *F. rossiae* SAF51 was strongly inhibited. Under these conditions *L. fabifermentans* SAF13 takes over *F. rossiae* SAF51.

When grown in association with the strain *A. pasteurianus* DSM 3509, *F. rossiae* SAF51 increased of about 4 log cycles its growth and had no effect on the growth of the *A. pasteurianus* strain. The same results were observed when *L. fabifermentans* SAF13 was grown in association with the *A. pasteurianus* strain. In this case the growth of *A. pasteurianus* incremented of about 1 log cycle, in relation to the presence of the strain *L. fabifermentans* SAF13.

## DISCUSSION

In this research we characterized the microbial population of the Dominican cocoa fermentation, with particular regards to LAB strains, from which we selected the minority strains *L. fabifermentans* SAF13 and *F. rossiae* SAF51 whose potential role in fermentation was investigated. In order to add to the body of knowledge on these poorly characterized species, we studied phenotypical attributes of interest, such as the ability of these strains to grow at low pH, high temperatures and high osmotic pressure, as the most important stressors of fermenting cocoa. In comparison to a cocoa related strain of the most representative species, *L. plantarum* B7, *L. fabifermentans* SAF13 behaved in a similar fashion in these conditions, demonstrating a good ability to survive and grow at low pH and high temperature, up to 42°C. *F. rossiae* SAF51, on the other hand, grew less efficiently in all conditions except in 150 g L^−1^ of sugar added medium, the approximate sugar content of cocoa pulp (De Vuyst & Weckx, 2016), in which this strain not only performed better than the other two strains, but grew more efficiently than in standard MRS medium (20 g L^−1^ glucose). Furthermore, the optimal pH for the growth of this strain was 5, lower than the strains of *L. plantarum* B7 and *L. fabifermentans* SAF13 that grew better at pH 6. This demonstrates the good adaptation that the strain of *F. rossiae* SAF51 has for conditions like those of fermenting cocoa: indeed, in cocoa pulp its growth after 40 h was comparable to *L. fabifermentans* SAF13 and to the growth in standard MRS medium.

The two strains behaved differently during the fermentation of glucose, fructose and sucrose, the main fermentable sugars present in cocoa pulp (Afoakwa et al., 2013). *L. fabifermentans* SAF13, similarly to *L. plantarum* B7, grew efficiently using all three substrates, whereas *F. rossiae* SAF51 had significantly longer lag times when growing in glucose and fructose, and, as reported by Corsetti et al. (2005), the species cannot ferment sucrose. The inability to ferment sucrose makes this species adequate for co‐culture growth as it reduces the competition for the carbon source. The production of lactic and acetic acid when fermenting glucose reflected their metabolic nature, as facultative heterofermentative *L. plantarum* and *L. fabifermentans* mainly produced lactic acid, whereas strictly heterofermentative *F. rossiae* produced a significantly higher amount of acetic acid. Strictly heterofermentative LAB are known to dominate the later stages of cocoa fermentation, mainly due to their higher resistance to stress factors and higher efficiency in ATP production (Gänzle, 2015).

Despite some beneficial activities have been proposed, generally the growth of mould in cocoa is considered non beneficial for the quality of the chocolate. Their growth during the pre‐processing stages of chocolate production, mainly during drying and storage, poses a significant risk due to the production of mycotoxins, mainly ochratoxin A and aflatoxin (Copetti et al., 2013). Given the stable nature of these compounds, prevention becomes key to avoiding their presence in the production chain. The ability of the strains of *F. rossiae* SAF51, and especially *L. fabifermentans* SAF13 to inhibit the growth of aflatoxinogenic species *A. flavus*, as well as other toxin‐producing *Aspergillus* species isolated from cocoa makes these strains good candidates for the biocontrol of aflatoxin‐producing mould species. These mould species occur commonly in tropical and subtropical regions; their presence during cocoa fermentation, especially in the last days of fermentation, has been documented in different geographical areas (Copetti et al., 2014; Delgado‐Ospina et al., 2021). From the literature, strains belonging to *A. tamarii* have been indicated to being producers of aflatoxins (Klich et al., 2000), whereas *A. nidulans* produces sterigmatocystin, a toxic precursor of aflatoxins (Delgado‐Virgen & Guzman‐de‐Peña, 2009).

The potential ability of *L. fabifermentans* SAF13 and *F. rossiae* SAF51 to produce cocoa‐specific aroma precursors by adding to the enzymatic pool of the fermentation process and increasing the amount of free amino acids that can be further metabolized (De Vuyst & Leroy, 2020) has been studied by evaluating their proteolytic activity at pH 4.5. Although proteolytic activity of LAB is generally studied at neutral pH (El Soda & Desmazeaud, 1982) we decided to stay closed to the pH of the cocoa beans during fermentation, in order to obtain results having a straightforward correlation with the real system. Moreover, the activity was studied on the cellular lysate. *L. fabifermentans* SAF13 shows an overall proteolytic activity slightly higher than *L. plantarum*, including the peptidase activity essential to release free amino acids. On the contrary, *F. rossiae* SAF51 has the lower specific activity on most of the substrate, but shows good endoprotease trypsin‐like activity and high peptidase activity on the hydrophobic amino acids Leucine. It is worth noting that at this acidic pH, all the three LAB show interesting endoprotease activity, that could boost the peptidase activity by producing new amino‐termini. The autolytic phenotype of *F. rossiae SAF51* is an added advantage to this activity as it can carry on after the initial stages of fermentation when LAB are dominant.

Finally, the data obtained indicated a good adaptability of the strains to growth in association, with a slightly limited yeast growth, which could indicate a competition for nutrients, rather than an inhibition of the yeast growth by the two LAB strains. The observed inhibition of the growth of *F. rossiae* is presumably in relation to the higher lag time observed for the strain. Another possible cause, which deserves to be deepened, is the ability of *L. fabifermentans* to produce bacteriocin‐like proteins, capable of inhibiting or delaying the growth of *L. rossiae*. In fact, Campanaro et al. (2014) previously observed that the sequenced genomes of *L. fabifermentans* present in the NCBI database contain a series of genes related to bacteriocin production and bacteriocin resistance. This inhibition was not observed when *A. pasteurianus* was added in association. The presence of the selected LAB strains did not inhibit the *A. pasteurianus* growth. This is a positive tract, because Acetic Acid Bacteria are fundamental in cocoa fermentation: oxidation of alcohol into acetic acid allows enzymatic and non‐enzymatic conversions inside the cocoa beans, providing the specific flavour precursor molecules.

In conclusion, the two strains *L. fabifermentans* SAF13 and *F. rossiae* SAF51 showed a high level of adaptation to the cocoa matrix, had a good resistance to stress factors related to the fermentation and possessed interesting activities that can be beneficial in a controlled cocoa fermentation process.

Further studies are in progress to evaluate the impact of adding these strains in a guided fermentation of cocoa on fermentation parameters and flavour profile of fine cocoa and chocolate.

## CONFLICT OF INTEREST

No conflict of interest declared.
